# Efficacy of intermittent epidural dexamethasone bolus for zoster-associated pain beyond the acute phase

**DOI:** 10.7150/ijms.46038

**Published:** 2020-07-11

**Authors:** Eun Mi Choi, Mi Hwa Chung, Joo Hyun Jun, Eun Hee Chun, In-Jung Jun, Jong Hee Park, Eun-ha Choi, Jung Eun Kim

**Affiliations:** Department of Anesthesiology and Pain Medicine, Kangnam Sacred Heart Hospital, Hallym University College of Medicine, Seoul, Republic of Korea

**Keywords:** continuous epidural infusion, dexamethasone, epidural, local anesthetics, neuropathic pain, zoster-associated pain

## Abstract

Herpes zoster develops when latent varicella zoster virus is reactivated in the trigeminal or dorsal root ganglions. Zoster-associated pain (ZAP) is neuropathic pain caused by the herpes zoster virus. Histological studies of postherpetic neuralgia patients suggest that inflammation is involved in ZAP. The effectiveness of local anesthetic and steroid epidural injections in ZAP patients has been reported. However, most studies included patients with acute herpes zoster, and the safety and therapeutic effects of different doses of epidural steroids in ZAP patients remain elusive. In this study, we randomly assigned 42 patients with severe ZAP beyond the acute phase, as determined by a numeric rating scale (NRS) score ≥7, to receive continuous epidural infusion of local anesthetics with either a one-time 5-mg dose or intermittent repeated doses (15 mg total) of dexamethasone. We found that intermittent repeated epidural dexamethasone bolus resulted in reduced NRS scores and an increased likelihood of complete remission in ZAP patients without any adverse effects. Thus, our results suggest that intermittent repeated epidural dexamethasone administration is safe and effective for treatment of ZAP beyond the acute phase.

## Introduction

Herpes zoster is caused by the reactivation of latent varicella zoster virus in the trigeminal and dorsal root ganglion 1,2. Zoster-associated pain is neuropathic pain resulting from peripheral nerve damage caused by the varicella zoster virus 3. Zoster-associated pain can reduce one's quality of life due to severe physical, psychological, social, and functional disturbances as consequences of chronic pain 4-7.

Herpes zoster-associated neuropathic pain is the consequence of inflammatory neural damage secondary to virus reactivation 8, which destroys the affected central and peripheral nerves and leads to inflammation and an immune response. This is proposed to involve two processes: sensitization and deafferentation. Peripheral and central sensitization result from nerve damage-induced excitability of primary afferent neurons, inflammation, and nociceptor irritation, whereas deafferentation is caused by degeneration of nociceptive neurons and dorsal root reorganization because of a declining number of C-fibers and the sprouting of Aβ fibers 9. These mechanisms suggest that appropriate analgesic and anti-inflammatory therapy may relieve postherpetic neuralgia (PHN) symptoms.

PHN is the most common complication of herpes zoster. Despite the existence of many treatments, PHN remains difficult to manage and is refractory to most treatment modalities 10-12. Previous studies have reported the effectiveness of epidural injections of local anesthetics and corticosteroids to control neuropathic pain in patients with zoster-associated pain 13-16. Epidural anesthetics delivered to the distal portion of the spinal cord block central sensitization and provide pain relief, and epidural steroids reduce inflammatory responses at the injured dorsal root ganglion, spinal cord, and surrounding peripheral tissue. However, most previous studies involved patients with acute herpes zoster, and few evaluated the effectiveness of epidural injections of local anesthetics and steroids in patients with PHN. Furthermore, no prior study has evaluated the therapeutic effects and safety of different doses of epidural steroids in patients with zoster-associated pain.

In this study, we conducted a randomized controlled trial to compare the analgesic and therapeutic effects of one-time administration of 5 mg epidural dexamethasone with intermittent repeated total 15-mg epidural dexamethasone. We evaluated the efficacy and safety of continuous epidural infusions of local anesthetics with dexamethasone and studied the efficacy of intermittent epidural dexamethasone bolus for zoster-associated pain beyond the acute phase.

## Materials and Methods

### Study design and patient population

The Hallym University Kangnam Sacred Heart Hospital institutional review board approved the protocol for this prospective, double-blind, randomized controlled trial (reference number 2019-04-011), and the trial was registered at ClinicalTrials.gov (NCT03995563, Principal investigator: J.E.K) prior to patient enrollment. Each participant provided written informed consent before undergoing any study-associated procedures. The study was conducted from June 2019 to February 2020 and included patients admitted to our pain clinic for zoster-associated pain rated as ≥ 7 on a numeric rating scale (NRS). We included only those patients who were beyond the acute phase of herpes zoster: specifically, 30 to 180 days after onset of the zoster rash. Patients were excluded if they met any of these criteria: 1) past or present conditions that may influence study participation, safety, or interpretation of the study results, such as cancer, severe diabetes, or a psychological, neurologic, liver, cardiac, hematologic, genitourinary, muscular, or dermatologic disorders; 2) current immunocompromised state; 3) herpes zoster involving the trigeminal nerve; 4) previous epidural block treatment for zoster-associated pain 5) epidural catheter discontinued before completing 10 days of continuous epidural infusion; 6) hemostatic disorder or receiving antiplatelet therapy; and 7) not an appropriate study participant, according to the opinion of the investigators. The study process was explained to all patients, and participants were instructed how to use the NRS for pain (ranging from 0 = no pain to 10 = worst pain imaginable). All participants received oral medications consisting of anticonvulsants, amitriptyline hydrochloride, and analgesics, as needed. Doses of oral medications were adjusted for age and renal function, and the drugs were tapered according to symptoms.

### Randomization

We used a computer-generated randomization schedule (www.randomizer.org) to generate a random allocation sequence. Patients were assigned at random in a 1:1 ratio to 1 of 2 groups, which differed according to whether a single dose or repeated doses of dexamethasone were administered. After patients entered the procedure room, another investigator (who had no other role in the conduct of the study) opened a sealed, opaque envelope containing the patient's group assignment. The patients were unaware of the group to which they were assigned.

**Figure 1:**
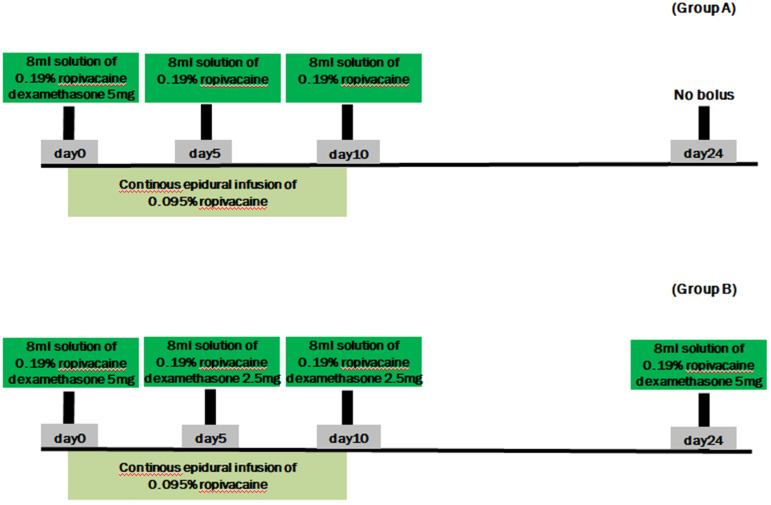
Summary of drug boluses. Group A received a continuous epidural infusion of 0.095% ropivacaine with a one-time 5-mg dexamethasone and 0.19% ropivacaine bolus. Group B received a continuous epidural infusion of 0.095% ropivacaine with intermittent repeated boluses of dexamethasone (total of 15 mg) and 0.19% ropivacaine.

After continuous epidural catheter placement, patients in group A received a continuous epidural infusion of local anesthetic and a one-time bolus of 5 mg dexamethasone and 0.19% ropivacaine; patients in group B received a continuous epidural infusion of local anesthetic and intermittent repeated boluses of dexamethasone and 0.19% ropivacaine, to a total of 15 mg dexamethasone (Figure 1). The study medication boluses were prepared in the procedure room by an anesthesiology resident who was not involved with the study. The resident used a table containing the instructions for preparing each 8-mL solution. The labels of all bolus syringes were hidden so neither the patients nor any of the medical staff in direct contact with the patients knew their contents. Outcomes were analyzed by a biostatistician who did not participate in the study in any other way and who knew nothing about the study except its design.

### Procedure

#### Group A: One-time 5-mg dexamethasone administration

All patients received 1 g cefazolin intravenously prior to the procedure and assumed the prone position on the procedure table. Under fluoroscopic guidance, an 18-gauge Tuohy needle was advanced into the epidural space at the second or third vertebral level below the target level. The target level was identified based on the affected dermatomal distribution of rash and pain. A 20-gauge epidural catheter (EpiStim^TM^ Sewoon Medical Co., Ltd., Seoul, Korea) was inserted through the Tuohy needle into the epidural space, and contrast medium was injected to identify placement of the catheter tip in the area of the affected spinal nerve. Then we have confirmed that the level to be targeted was correlated to the affected dermatome by electrical nerve stimulation. After injection of an initial bolus of 0.19% ropivacaine and 5 mg dexamethasone (total of 8 mL), a continuous epidural infusion of ropivacaine (275 mL, 0.095%) was begun at a rate of 7 mL/h using the AutoFuser pump (ACE Medical Co., Ltd., Seoul, Korea), which is a portable balloon infusion device. The catheter was fixed in place with subcutaneous tunneling to minimize infection risk and performed daily dressing changes at the tunneling site. The continuous epidural infusion was maintained for 10 days. During the 10-day period, patients in group A received an 8-mL bolus of 0.19% ropivacaine through the epidural catheter every 5 days. All patients (in groups A and B) were monitored in the recovery room for 30 minutes after each bolus injection.

#### Group B: Intermittent repeated total 15-mg dexamethasone administration

Patients in group B also received 1 g cefazolin intravenously before the procedure and assumed a prone position on the procedure table. As with group A, an 18-gauge Tuohy needle was advanced into the epidural space, through which a 20-gauge epidural catheter was inserted into the epidural space to reach the affected spinal level. After confirming the position of the catheter tip using C-arm imaging and electrical nerve stimulation, an initial bolus injection of 0.19% ropivacaine and 5 mg dexamethasone (total of 8 mL) was administrated through the epidural catheter. Similar to group A, a continuous epidural infusion of ropivacaine (275 mL, 0.095%) was begun at 7 mL/h using the AutoFuser pump and maintained for 10 days. Unlike group A, patients in group B received 0.19% ropivacaine and 2.5 mg dexamethasone (total of 8 mL) via the epidural catheter every 5 days over the 10-day infusion period. They also received a final epidural injection of 0.19% ropivacaine and 5 mg dexamethasone (total of 8 mL) 2 weeks after the epidural catheter was removed (Figure 1).

### Outcome assessments

A staff anesthesiologist who was blinded to the patients' group assignments collected the following data: sex, age, level of the involved dermatome, time from onset of herpes zoster rash to epidural infusion, and presence of diabetes, hypertension, or chronic obstructive pulmonary disease.

The primary outcome measure was change in NRS pain score. We assessed whether pain scores significantly declined during the first 6 months after the procedure and calculated the percentage of patients with ≥ 50% reduction in pain severity (NRS scores) after treatment in each group. NRS pain scores and the percentage of patients with ≥ 50% reduction in severity of pain were compared between the two groups before and at monthly intervals from 1 to 6 months after the treatment. The secondary outcome was complete remission of PHN. Complete remission was defined as an NRS pain score ≤ 2, resolved allodynia and hyperalgesia, and no longer requiring medical treatment. To assess the presence of allodynia or hyperalgesia, we performed a touch/pressure test using a filament brush, a pinprick test using a 26G needle, and a cold swab test using alcohol-soaked cotton. The percentage of patients in each group with complete remission of zoster-associate pain was calculated and compared between groups.

### Statistical analysis

Kolmogorov-Smirnov test was used to evaluate the normality of demographic data. Continuous variables are reported as mean ± standard deviation and were analyzed using independent t-tests. Categorical variables are reported as frequency and percentage and were analyzed using Chi-square or Fisher's exact tests. We used logistic regression models to estimate the odds of a ≥ 50% reduction in pain severity (NRS scores) or complete remission of PHN within 6 months of treatment.

Based on our experience and data from a pilot study, the sample size was calculated with G*Power software (version 3.0.10) using the z test with a p1 proportion of 0.9, p2 proportion of 0.5, error probability of 0.05, and power of 0.8. Using these parameters, we determined that 20 patients were required in each group to detect a sufficient effect size. We therefore designed the study with 22 patients per group, assuming a 10% drop-out rate. Data were analyzed using SPSS software (version 23, IBM Corp., Armonk, NY, USA) or SAS (version 9.4, SAS Inc., Cary, NC, USA), and P values < 0.05 were deemed statistically significant.

## Results

**Figure 2:**
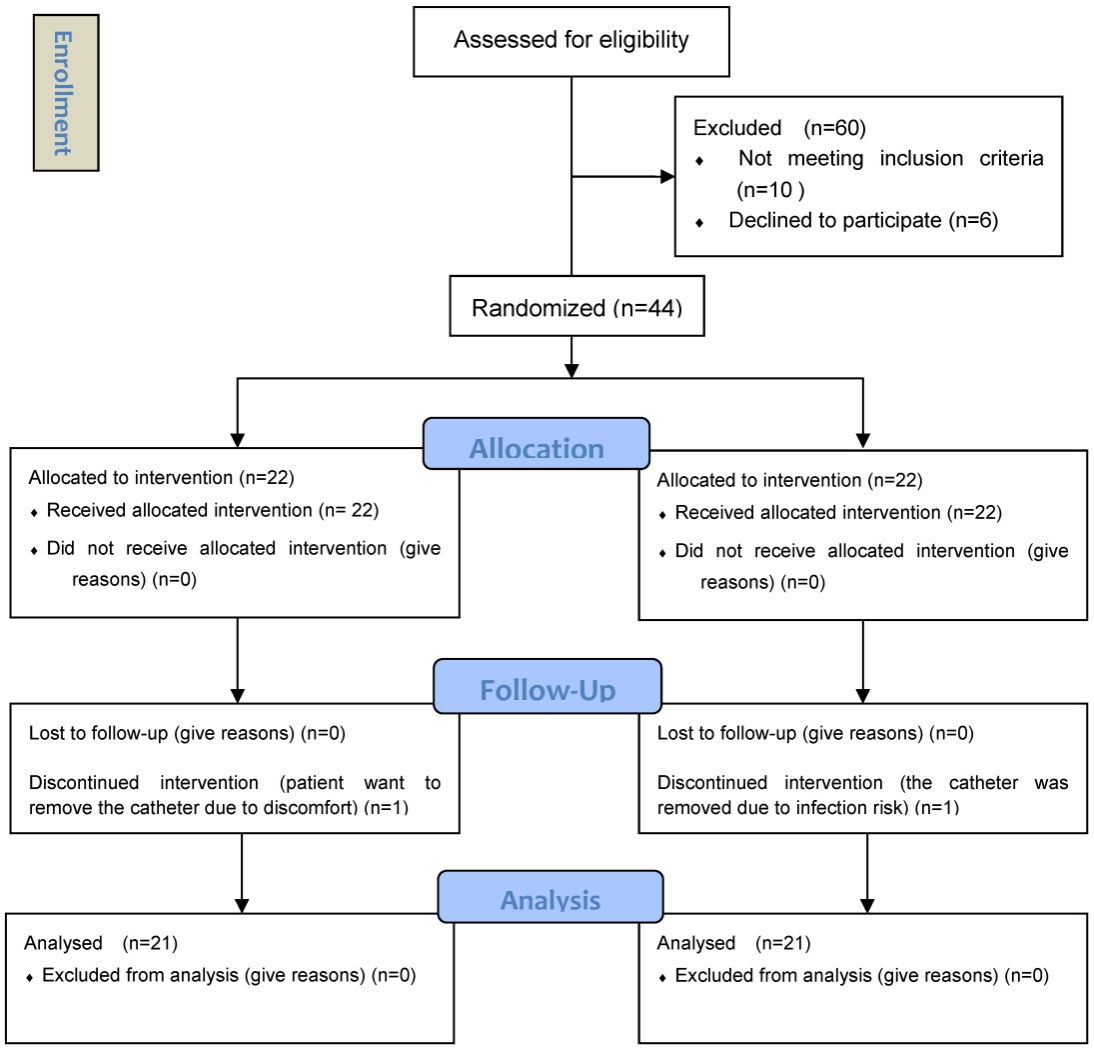
CONSORT flow diagram.

A total of 60 patients were screened between April 2019 and February 2020: 10 failed to meet the inclusion criteria, and 6 did not agree to participate. Consequently, 44 patients were randomized (22 per group), and 42 were included in the final analysis (as shown in Figure 2). Age, sex, presence of underlying disorders, level of involved dermatome, time from rash onset to continuous epidural infusion, and pre-treatment NRS scores were similar between groups A and B (Table 1).

**Figure 3:**
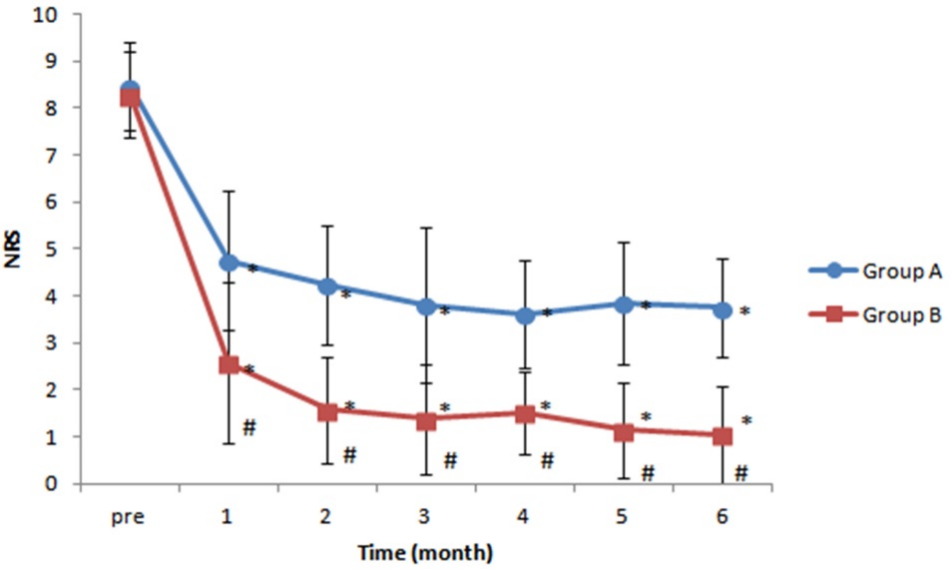
Changes in numeric rating scale (NRS) pain scores over time. Patients in both groups exhibited significant reductions in NRS scores at all time points after treatment, compared with the pre-treatment value, but patients in group B (intermittent repeated total 15-mg dexamethasone administration group) had significantly lower NRS scores than patients in group A (one-time 5-mg dexamethasone administration group) at all post-treatment times. *P < 0.05 versus pre-treatment NRS score. #P < 0.05 versus group A.

NRS pain scores were lower after treatment than before treatment in both groups' at all follow-up times. When post-procedure pain scores were compared between groups, patients in group B (intermittent repeated total 15-mg dexamethasone administration) exhibited significantly lower pain scores than patients in group A (one-time 5-mg dexamethasone administration) at each month after treatment (P < 0.05) (Figure 3).

The percentage of patients with ≥ 50% reduction in pain severity was not significantly different between groups (P = 0.110). However, the percentage of complete remission of PHN was significantly higher in group B than in group A (28.57% in group A vs 80.95% in group B, P = 0.001; odds ratio [OR] 10.63, 95% confidence interval [CI] 2.51-44.98) (Table 2).

No serious procedure-related complications were detected in either group A or group B. Specifically; no patient developed an epidural hematoma, infection, or abscess.

## Discussion

In this randomized, controlled trial, continuous epidural local anesthetic infusion with intermittent repeated dexamethasone administration provided excellent analgesia for herpes zoster-associated pain, allodynia, and hyperalgesia beyond the acute phase. Pain relief persisted during the 6 months of follow-up and was not accompanied by adverse effects.

The beneficial effects of epidural dexamethasone have been attributed to interruption of neurogenic inflammation and promotion of nerve tissue repair by reducing edema, as well as cytotoxic responses 17-19. In addition, epidural local anesthetic infusion blocks nociceptive input at distal portion of the spinal cord (i.e., in the dorsal root ganglion, spinal nerve roots, and peripheral regions of the cord), providing analgesia and interrupting sensitization 20-23. Through these mechanisms, continuous epidural infusion of local anesthetic and dexamethasone reduced pain in both of our study groups, compared with before treatment, and effectiveness was greater in patients who received intermittent epidural dexamethasone bolus.

Although the pathogenesis of PHN is not fully understood, herpes zoster is known to affect the central and peripheral nervous systems, resulting in PHN 24,25. PHN-related neuropathic pain is caused by inflammatory neural damage produced by the varicella zoster virus 26. Two mechanisms of PHN have been proposed, including sensitization and deafferentation 27,28. Sensitization is caused by loss of γ-aminobutyric acid inhibitory neurons in the dorsal root ganglion after nerve injury, as well as reduction of the nociceptor threshold induced by inflammatory mediators, such as histamine, substance P, and various cytokines 29,30. This sensitization causes spontaneous pain and hyperalgesia. Deafferentation is caused by dorsal root reorganization, resulting from a decline in the number of C fibers and the sprouting of Aβ fibers 27,29, which rewire in the dorsal root ganglion and connect with the pain-transmitting spinothalamic tract, thereby producing allodynia and paresthesias. These mechanisms suggest that appropriate analgesic and anti-inflammatory therapy could relieve the symptoms of PHN.

Histopathologic studies in humans indicate that inflammatory processes may be involved in the development of PHN. In a postmortem study, Watson et al. reported that infiltration and accumulation of immune cells occurred around the spinal cord and nerves of patients with PHN 31. This inflammatory component of PHN suggests that anti-inflammatory treatment may reverse or at least limit progression of the disorder. Corticosteroids act directly on membranes to prevent ectopic discharges from C fibers and provide analgesia by inhibiting the transmission of nociceptive input 32-34. As potent inhibitors of inflammation, steroids also suppress concomitant edema-induced ischemic nerve damage and reduce deafferentation processes 35,36.

Previous studies have shown that intrathecal methylprednisolone relieves PHN-related pain and allodynia 11, 37-39. In these studies, intrathecal methylprednisolone was associated with reduced cerebrospinal fluid concentrations of interleukin-8, suggesting that this treatment reduced pain and allodynia by decreasing an ongoing inflammatory reaction. However, intrathecal methylprednisolone produced serious complications, including chemical or aseptic meningitis, cauda equina syndrome, transverse myelitis, lumbar radiculitis, chronic arachnoiditis, urinary retention, and intractable headaches 40,41. Neurotoxicity from intrathecal corticosteroids has been ascribed not only to the steroid but also to co-delivered local anesthetics, hyperbaric mixtures, or preservatives (e.g., polyethylene glycol, benzalkonium chloride, benzyl alcohol) in the steroid solution 42. Currently, methylprednisolone is not approved for intrathecal administration because of its severe adverse effects, including arachnoiditis 43.

In this study, we used continuous infusion of dexamethasone administered epidurally, instead of via the intrathecal route, in patients with zoster-associated pain beyond the acute phase. This treatment reduced NRS pain scores in all 42 patients in our study, and intermittent repeated administration of dexamethasone was more effective in alleviating zoster-associated pain, allodynia, and hyperalgesia than one-time dexamethasone administration. The herpes zoster viral burden settles in the dorsal root ganglion of the affected segment, and it produces nerve and tissue damage. Epidural drug administration targeting the affected spinal root applies the drug directly at the area of the pathologic nerve and effectively controls zoster-associated pain, while avoiding systemic adverse effects and neurotoxicity. There have been no reports of serious complications with epidural steroid administration for zoster-associated pain, unlike the serious adverse effects reported with intrathecal methylprednisolone.

There are some limitations in the current study. There was no control group of patients who received no epidural steroids. Because previous reports indicated that neuropathic pain associated with herpes zoster arises from inflammatory neural damage and that epidural steroids and local anesthetics are highly effective for acute herpes zoster pain 13,14,21, we considered a control group of patients receiving an epidural infusion of local anesthetic alone (without corticosteroid) to be unethical. Another limitation was that we enrolled patients 30 to 180 days after onset of the herpes zoster rash and therefore did not include patients with “well-established” PHN. Dworkin et al. defined herpes zoster-related pain persisting longer than 6 months after rash onset as “well-established PHN” 44. However, pain persisting > 180 days after rash onset has a low likelihood of improving, and patients with “well-established” PHN are unlikely to experience reductions in pain or complete remission 45. Thus, we designed the current study to evaluate the effectiveness of intermittent epidural dexamethasone bolus in patients with zoster-associated pain beyond the acute phase, while excluding those with “well-established” PHN. We plan to include patients with established PHN in future studies investigating the effectiveness of epidural corticosteroids.

In conclusion, continuous epidural infusion of local anesthetics with intermittent repeated dexamethasone administration resulted in a greater reduction in pain and likelihood of complete remission than one-time bolus of dexamethasone.

## Figures and Tables

**Table 1:** Patient characteristics in both groups. Baseline characteristics were not significantly different between groups. Group A: one-time 5-mg dexamethasone administration group; Group B: intermittent repeated total 15-mg dexamethasone administration group; NRS, numeric rating scale; SD, standard deviation.

	Group A (n = 21)	Group B (n = 21)	P-value
Age (years), mean ± SD	64.67 ± 13.06	67.43 ± 9.85	0.444
Sex, (male/female) n	7/14	10/11	0.346
**Presence of underlying disease, n (%)**			
None	9 (42.86)	9 (42.86)	> 0.999
Hypertension, n (%)	12 (57.14)	10 (47.62)	
Diabetes mellitus, n (%)	3 (14.29)	4 (19.05)	
Chronic obstructive pulmonary disease, n (%)	2 (9.52)	2 (9.52)	
**Level of involved dermatome, n (%)**			
Cervical	1 (4.76)	2 (9.52)	0.740
Thoracic	16 (76.19)	17 (80.95)	
Lumbar	4 (19.05)	2 (9.52)	
Time from rash onset to continuous epidural infusion (weeks), mean ± SD	10.95 ± 7.29	10.14 ± 5.87	0.694
Pre-treatment NRS score, mean ± SD	8.48 ± 0.93	8.29 ± 0.90	0.504

**Table 2:** Between-group comparisons of reduction in pain severity and complete remission. Complete remission was defined as an NRS score ≤ 2, resolved allodynia and hyperalgesia, and no longer requiring medical treatment. Data were analyzed by logistic regression analysis. The odds of ≥ 50% reduction in pain severity and complete remission were 6.25-times and 10.625-times higher, respectively, for group B than for group A. Group A: one-time 5-mg dexamethasone administration group; Group B: intermittent repeated total 15-mg dexamethasone administration group; OR, odds ratio; CI, confidence interval; NRS, numeric rating scale.

	Reduction in pain severity				Complete remission			
	≥ 50% reduction in pain severity	< 50% reduction in pain severity	OR (95% CI)	P-value	NRS score ≤ 2, resolved allodynia and hyperalgesia	NRS score > 2, persisted allodynia and hyperalgesia	OR (95% CI)	P-value
**Group A, n (%)**	16 (76.19)	5 (23.81)	Reference		6 (28.57)	15 (71.43)	Reference	
**Group B, n (%)**	20 (95.24)	1 (4.76)	6.25 (0.66-59.03)	0.110	17 (80.95)	4 (19.05)	10.63 (2.51-44.98)	0.001

## References

[1] Hillebrand K, Bricout H, Schulze-Rath R, Schink T, Garbe E (2015). Incidence of herpes zoster and its complications in Germany, 2005-2009. J Infect.

[2] Kim YJ, Lee CN, Lim CY, Jeon WS, Park YM (2014). Population-based study of the epidemiology of herpes zoster in Korea. J Korean Med Sci.

[3] Gershon AA (1996). Epidemiology and management of postherpetic neuralgia. Semin Dermatol.

[4] Katz J, Cooper EM, Walther RR, Sweeney EW, Dworkin RH (2004). Acute pain in herpes zoster and its impact on health-related quality of life. Clin Infect Dis.

[5] Gater A, Abetz-Webb L, Carroll S, Mannan A, Serpell M, Johnson R (2014). Burden of herpes zoster in the UK: findings from the zoster quality of life (ZQOL) study. BMC Infect Dis.

[6] Johnson RW, Bouhassira D, Kassianos G, Leplege A, Schmader KE, Weinke T (2010). The impact of herpes zoster and post-herpetic neuralgia on quality-of-life. BMC Med.

[7] Serpell M, Gater A, Carroll S, Abetz-Webb L, Mannan A, Johnson R (2014). Burden of post-herpetic neuralgia in a sample of UK residents aged 50 years or older: findings from the Zoster Quality of Life (ZQOL) study. Health Qual Life Outcomes.

[8] Kim H, Choi YL, Lee DK, Choi SS, Lee IO, Kong MH (2012). The neurological safety of intrathecal acyclovir in rats. Pain Physician.

[9] Woolf CJ, Mannion RJ (1999). Neuropathic pain: aetiology, symptoms, mechanisms, and management. Lancet.

[10] Milligan NS, Nash TP (1985). Treatment of post-herpetic neuralgia. A review of 77 consecutive cases. Pain.

[11] Lin CS, Lin YC, Lao HC, Chen CC (2019). Interventional Treatments for Postherpetic Neuralgia: A Systematic Review. Pain Physician.

[12] Johnson RW, McElhaney J (2009). Postherpetic neuralgia in the elderly. Int J Clin Pract.

[13] Opstelten W, Van Wijck AJ (2004). The pine study: Rationale and designe of a randomised comparison of epidural injection of local anaestheteics and steroids versus care-as-usual to prevent postherpetic neuralgia in the elderly. BMC Anesthesiol.

[14] Van Wijck AJ, Opstelten W (2006). The pine study of epidural steroids and local anaesthetics to prevent postherpetic neuralgia: A randomised controlled trial. Lancet.

[15] Pasqualucci A, Pasqualucci V, Galla F (2000). Prevention of post-herpetic neuralgia: Acyclovir and prednisolone versus epidural local anaesthetic and methylprednisolone. Acta Anaesthesiol Scand.

[16] Seo YG, Kim SH, Choi SS (2018). Effectiveness of continuous epidural analgesia on acute herpes zoster and postherpetic neuralgia: A retrospective study. Medicine (Baltimore).

[17] Hall ED (1982). Glucocorticoid effects on central nervous excitability and synaptic transmission. Int Rev Neurobiol.

[18] Forrest JB (1980). The response to epidural steroid injections in chronic dorsal root pain. Can Anaesth Soc J.

[19] Marshall LL, Trethewie ER, Curtain CC (1977). Chemical radiculitis. A clinical, physiological and immunological study. Clin Orthop Relat Res.

[20] Manabe H, Dan K, Higa K (1995). Continuous epidural infusion of local anesthetics and shorter duration of acute zoster-associated pain. Clin J Pain.

[21] Hwang SM, Kang YC, Lee YB, Yoon KB, Ahn SK, Choi EH (1999). The effects of epidural blockade on the acute pain in herpes zoster. Arch Dermatol.

[22] Riopelle JM, Naraghi M, Grush KP (1984). Chronic neuralgia incidence following local anesthetic therapy for herpes zoster. Arch Dermatol.

[23] Manabe H, Dan K, Hirata K, Hori K, Shono S, Tateshi S (2004). Optimum pain relief with continuous epidural infusion of local anesthetics shortens the duration of zoster-associated pain. Clin J Pain.

[24] Nurmikko T, Wells C, Bowsher D (1991). Pain and allodynia in postherpetic neuralgia: role of somatic and sympathetic nervous systems. Acta Neurol Scand.

[25] Kim ED, Lee YI, Park HJ (2017). Comparison of efficacy of continuous epidural block and pulsed radiofrequency to the dorsal root ganglion for management of pain persisting beyond the acute phase of herpes zoster. PLoS One.

[26] Johnson RW, Rice AS (2014). Clinical practice. Postherpetic neuralgia. N Engl J Med.

[27] Baron R (2008). Mechanisms of postherpetic neuralgia-we are hot on the scent. Pain.

[28] Johnson RW (2007). Zoster-associated pain: what is known, who is at risk and how can it be managed?. Herpes.

[29] van Wijck AJ, Wallace M, Mekhail N, van Kleef M (2011). Evidence-based interventional pain medicine according to clinical diagnoses. 17. Herpes zoster and post-herpetic neuralgia. Pain Pract.

[30] Premkumar LS (2010). Targeting TRPV1 as an alternative approach to narcotic analgesics to treat chronic pain conditions. AAPS J.

[31] Watson CP, Deck JH, Morshead C, Van der Kooy D, Evans RJ (1991). Post-herpetic neuralgia: further post-mortem studies of cases with and without pain. Pain.

[32] Devor M, Govrin-Lippmann R, Raber P (1985). Corticosteroids suppress ectopic neural discharge originating in experimental neuromas. Pain.

[33] Eaglstein WH, Katz R, Brown JA (1970). The effects of early corticosteroid therapy on the skin eruption and pain of herpes zoster. JAMA.

[34] Forrest JB (1978). Management of chronic dorsal root pain with epidural steroid. Can Anaesth Soc J.

[35] Winnie AP, Hartwell PW (1993). Relationship between time of treatment of acute herpes zoster with sympathetic blockade and prevention of post-herpetic neuralgia: Clinical support for a new theory of the mechanism by which sympathetic blockade provides therapeutic benefit. Reg Anesth.

[36] Fields HL, Rowbotham M, Baron R (1998). Postherpetic neuralgia: Irritable nociceptors and deafferentation. Neurobol Dis.

[37] Kumar V, Krone K, Mathieu A (2004). Neuraxial and sympathetic blocks in herpes zoster and postherpetic neuralgia: An appraisal of current evidence. Reg Anesth Pain Med.

[38] Kotani N, Kushikata T, Hashimoto H, Kimura F, Muraoka M, Yodono M (2000). Intrathecal methylprednisolone for intractable postherpetic neuralgia. N Engl J Med.

[39] Kikuchi A, Kotani N, Sato T, Takamura K, Sakai I, Matsuki A (1999). Comparative therapeutic evaluation of intrathecal versus epidural methylprednisolone for long-term analgesia in patients with intractable postherpetic neuralgia. Reg Anesth Pain Med.

[40] Nelson DA, Landau WM (2001). Intrathecal methylprednisolone for postherpetic neuralgia. N Engl J Med.

[41] Nelson DA, Landau WM (2002). Intrathecal steroid therapy for postherpetic neuralgia: a review. Expert Rev Neurother.

[42] Nelson DA (1993). Intraspinal therapy using methylprednisolone acetate. Twenty-three years of clinical controversy. Spine (Phila Pa 1976).

[43] Dubinsky RM, Kabbani H, El-Chami Z, Boutwell C, Ali H, Quality Standards Subcommittee of the American Academy of N (2004). Practice parameter: treatment of postherpetic neuralgia: an evidence-based report of the Quality Standards Subcommittee of the American Academy of Neurology. Neurology.

[44] Dworkin RH, Gnann JW Jr, Oaklander AL, Raja SN, Schmader KE, Whitley RJ (2008). Diagnosis and assessment of pain associated with herpes zoster and postherpetic neuralgia. J Pain.

[45] Fishman SM, Ballantyne JC, Rathmell JP (2010). Bonica's Management of Pain 4^th^ edition. Lippincott Williams & Wilkins, Philadelphia.

